# Novel transition metal-free synthetic protocols toward the construction of 2,3-dihydrobenzofurans: a recent update

**DOI:** 10.3389/fchem.2024.1470861

**Published:** 2024-12-13

**Authors:** Aqsa Mushtaq, Muhammad Irfan, Atta ul Haq, Asim Mansha, Samreen Gul Khan, Ameer Fawad Zahoor, Bushra Parveen, Ali Irfan, Katarzyna Kotwica-Mojzych, Mariola Glowacka, Mariusz Mojzych

**Affiliations:** ^1^ Department of Chemistry, Government College University Faisalabad, Faisalabad, Pakistan; ^2^ Department of Pharmaceutics, Government College University, Faisalabad, Pakistan; ^3^ Department of Basic Sciences, Department of Histology, Embriology and Cytophysiology, Medical University of Lublin, Lublin, Poland; ^4^ Faculty of Health Sciences Collegium Medicum, The Mazovian Academy in Plock, Płock, Poland

**Keywords:** dihydrobenzofurans, transition metal-free, photocatalytic, organocatalyzed, catalyst free

## Abstract

2,3-Dihydrobenzofurans are noteworthy scaffolds in organic and medicinal chemistry, constituting the structural framework of many of the varied medicinally active organic compounds. Moreover, a diverse variety of biologically potent natural products also contain this heterocyclic nucleus. Reflecting on the wide biological substantiality of dihydrobenzofurans, several innovative and facile synthetic developments are evolving to achieve these heterocycles. This review summarizes the transition-metal-free, efficient, and novel synthetic pathways toward constructing the dihydrobenzofuran nucleus established after 2020.

## 1 Introduction

**FIGURE 1:**
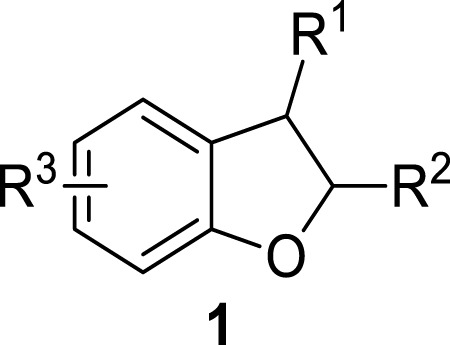
General structure of dihydrobenzofuran **1**.

Five-membered heterocycles and their derivatives exhibit a remarkable role in the pharmaceutical industry (Delost et al., 2018; Irfan et al., 2023a; Irfan et al., 2023b). 2,3-Dihydrobenzofurans are composed of a benzene ring attached to a dihydrofuran skeleton (oxygen involving a five-membered ring), which can easily be obtained as a result of hydrogenation of benzofurans (benzene attached to a furan ring). The 2,3-dihydrobenzofurans were formerly called “coumarane.” Dihydrobenzofurans are generally neutral or weakly basic. A diverse range of medicinally active organic compounds are known to include the dihydrobenzofuran ring and its derivatives in their structural framework, thereby emphasizing the biological potential of this moiety (Khanam, 2015; Naik et al., 2015; Sachdeva et al., 2022; Farooq et al., 2019). The general structure of dihydrobenzofurans has been presented in Figure 1 (Chen et al., 2019).

**FIGURE 2:**
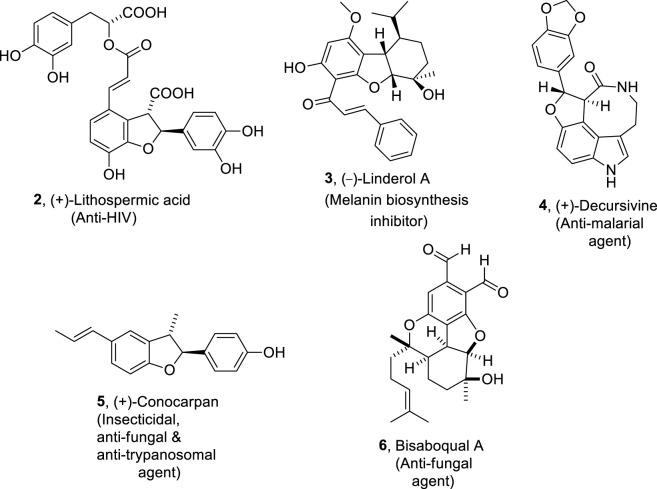
Structures of the dihydrobenzofuran ring constituting biologically active natural products.

Various naturally occurring organic compounds are also composed of 2,3-dihydrobenzofuran ring skeletons which play a significant role in medicinal chemistry (Chen et al., 2019; Macías et al., 1999; Pieters et al., 1999). Some of the significant biologically active natural products constituting dihydrobenzofuran core in their skeletal framework are (+)-lithospermic acid (Varadaraju and Hwu, 2012) **2**, (−)-linderol A (Mimaki et al., 1995; Yamashita et al., 2003) **3**, bisabosqual A (Minagawa et al., 2001; Snider and Lobera, 2004; am Ende et al., 2013) **6**, and (+)-decursivine (Sun et al., 2011) **4**, which are used against human immunodeficiency virus, inhibit the melanin’s biosynthesis, and treat fungal diseases and malarial infection, respectively. (+)-Conocarpan (Chen and Weisel, 2013; Zheng et al., 2003) **5** is another naturally occurring dihydrobenzofuran constituting organic compound known for its broad pharmaceutical applications, including anti-trypanosomal, insecticidal, and anti-fungal action (Figure 2). This unparalleled medicinal effectivity has resulted in several endeavors regarding the procurement of dihydrobenzofuran-based compounds.

The initial work on the synthesis of dihydrobenzofuran was reported in 1892. Since then, several methodologies have been presented concerning the construction of the dihydrobenzofuran skeleton. However, the synthetic routes developed in the early twentieth century were not found to be satisfactory in terms of yield and productivity. The developed protocols suffered from certain challenges, including steps involving the utilization of toxic and costly metal catalysts, harsh reaction conditions, and restricted substrate scope (Li et al., 2023). Most of the developed methods involve the use of pre-functionalized radicals as starting materials (Chen et al., 2023). Moreover, in a few instances, reactivity was observed to be diminished due to steric crowding (Lu et al., 2021). Over the years, researchers have proposed numerous novel, efficient, and high-yielding alternative synthetic pathways to achieve dihydrobenzofurans (Alexander, 1892; Rao et al., 2003).

Transition metal-catalyzed reactions have gained considerable significance owing to their employment in achieving several organic compounds (Pannilawithana et al., 2021; Bai et al., 2018). Among diversely reported efficient strategies for synthesizing dihydrobenzofurans and their derivatives, transition metal catalysis has also taken a significant locale in this domain. Various transition metal catalysts, including ruthenium (Sau et al., 2023), iridium (Kusaka et al., 2022), rhodium (Jiang et al., 2022), gold (Morita et al., 2023), nickel (Jin et al., 2022), palladium (Sun et al., 2023; Ding et al., 2023; Kang et al., 2022), platinum (Yang et al., 2023), vanadium (Wang et al., 2023), silver (Guo et al., 2023), tungsten (Gowda et al., 2023), and copper (Zhu et al., 2023; Rao and Islam, 2021), have been exploited to introduce the efficient catalytic systems for the construction of dihydrobenzofuran nucleus by treating different substrates. Individual and dual metal catalysis approaches have been demonstrated to afford high-yielding dihydrobenzofurans in a feasible manner (Huang et al., 2022; Zhao et al., 2023).

Apart from widely employed transition metal catalysis, several transition metal-free approaches regarding the synthesis of dihydrobenzofurans and their derivatives have been accomplished in recent years, which involved the utilization of visible light-promoted preparation (Caiuby et al., 2018), electrocatalysis (Kärkäs, 2018), and Bronsted (Suneja and Schneider, 2018) and Lewis (Sharma et al., 2017) acid-mediated synthetic routes to forge the dihydrobenzofuran nucleus. With respect to the increasing concerns regarding ever-increasing environmental pollution, catalyst-free synthesis has also gained definite significance as per green chemistry criteria (Chen et al., 2018).

Several reviews have been published highlighting the significance of dihydrobenzofurans and their derivatives. Bertolini and Pineschi (2009) reported a review on the development of benzofuran synthesis. In 2011, Sheppard provided an overview of newly developed synthetic pathways to achieve dihydrobenzofurans (Sheppard, 2011). In 2019, Chen et al. covered the synthesis of natural products involving dihydrobenzofuran rings in their structural formula (Chen et al., 2019). Afterward, in early 2020, a review covering the synthetic protocols developed in 2012–2019 was put forward by Laurita et al. (2020). Similarly, in 2022, a comprehensive review was published by Dapkekar et al. (2022), which focused on the newly developed synthetic routes to furnish the 2,3-dihydrobenzofurans and 1,3-dihydroisobenzofurans along with their applications. Recently, transition metal-catalyzed protocols to access dihydrobenzofuran nuclei have been reviewed by our group (Ashraf et al., 2024). Currently, developments are in progress regarding the construction of the dihydrobenzofuran skeleton. The focus of this review is to summarize the transition metal-free novel synthetic protocols toward the construction of dihydrobenzofurans developed during 2021–2023.

## 2 Review of the literature

### 2.1 Bronsted acid-induced synthesis of dihydrobenzofurans

**SCHEME 1:**
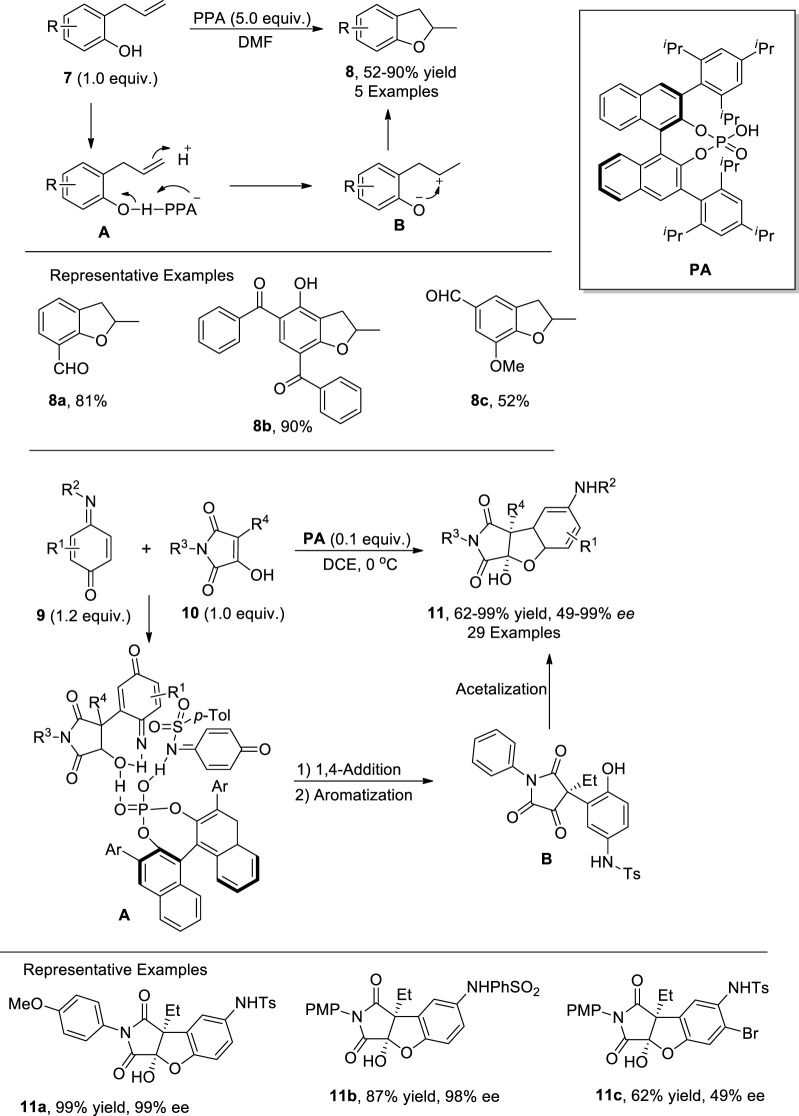
Synthesis of dihydrobenzofurans **8** and **11** using a Bronsted acid as the catalyst.

Since the beginning of the twenty-first century, Bronsted acids have been widely utilized as efficacious catalysts for the formation of C–C bonds as they activate the electrophilic centers for facile nucleophilic addition reactions (Yamamoto and Ishihara, 2008; Jacob et al., 2017). Yang et al. (2021) reported the Bronsted acid- [i.e., polyphosphoric acid (PPA)]-mediated synthesis of 2,3-dihydrobenzofuran derivatives **8**. In their novel approach, ortho-allyl/prenyl phenols **7** were transformed into desired cyclic heterocycles **8** via the activation of phenolic oxygen by PPA in dimethylformamide. The proposed mechanism involved the generation of phosphorylated adduct **A** by treating substituted phenols **7** with PPA at increased temperature. The disruption of electron stability in **A** gave rise to intermediate **B**, which subsequently led to the synthesis of dihydrobenzofurans **8** via nucleophilic annulation. The target molecules were obtained in moderate to excellent yields (52%–90%) by using the above-mentioned strategy (Scheme 1).

In the same year, another phosphoric acid (PA)-catalyzed synthesis of dihydrobenzofurans was documented by Zhang et al. (2021) by employing a [3 + 2] annulation reaction, which is widely carried out to construct sophisticated heterocyclic functionalities. They designed a facile methodology for the enantioselective preparation of 2,3-dihydrobenzofuran derivatives **11** via stereoselective [3 + 2] annulation. In their innovative methodology, substituted quinone monoimines **9** were reacted with 3-hydroxymaleimides **10** in the presence of asymmetric phosphoric acid catalyst (PA) in dichloroethane (DCE) to achieve moderate to excellent yields (62%–99%) of dihydrobenzofurans with remarkable enantioselectivities (49%–99% *ee*). The plausible reaction mechanism commenced with the activation of both reactants by PA, followed by the attack of 3-hydroxymaleimides **10** on quinone monoimines **9** to generate intermediate **A**, which further underwent 1,4-addition and aromatization to afford intermediate **B**. Upon intramolecular cyclization, the intermediate **B** furnished the desired succinimide fused 2,3-dihydrobenzofurans **11** (Scheme 1) (Zhang et al., 2021).

**SCHEME 2:**
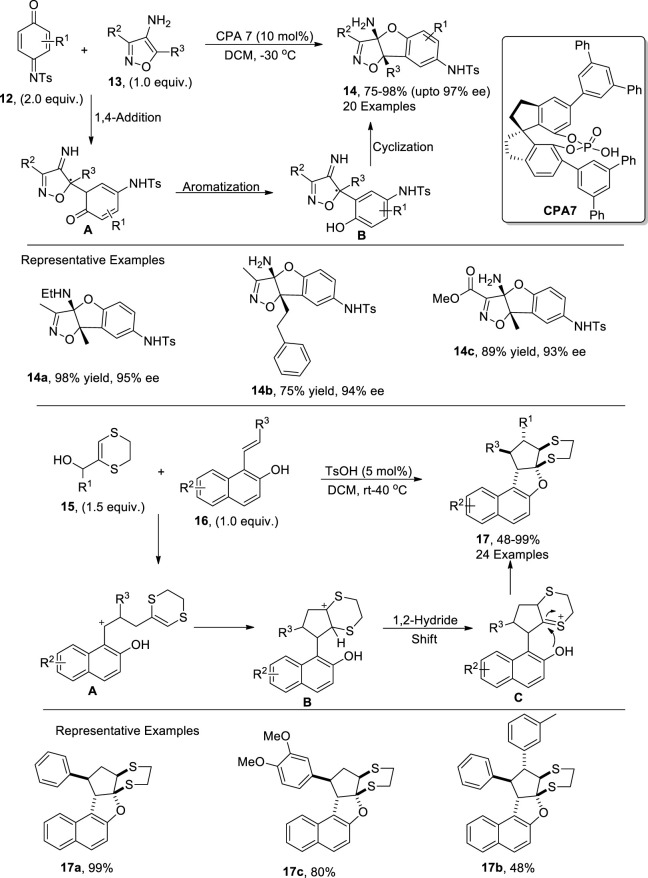
Synthesis of dihydrobenzofurans **14** and **17** using a Bronsted acid as the catalyst.

In order to overcome microbial diseases, synthetic researchers have carried out several synthetic efforts to afford efficient antimicrobial agents (Breijyeh and Karaman, 2023; Zahoor et al., 2017). Yan et al. (2022) demonstrated another productive enantioselective route to synthesize dihydrobenzofurans **14** via a chiral phosphoric acid-catalyzed [3 + 2] annulation reaction between quinone imines **12** and 4-aminoisoxazoles **13** by utilizing dichloromethane as solvent. The reaction mechanism was believed to involve a 1,4-addition reaction followed by an aromatization reaction to yield intermediate **B**. The intermediate **B** was then subjected to cyclization to afford target isoxazoline-based-dihydrobenzofurans **14** in efficient yields. The resulting dihydrobenzofurans **14** were then found to be effective against disease-causing fungi in plants (Scheme 2).

Li et al. (2023) also discussed the Bronsted acid (*p*-toluene sulfonic acid)-mediated [3 + 2] cycloaddition reaction involving the synthesis of dihydrobenzofurans. The newly developed efficient synthetic protocol involved the treatment of substituted styrylnaphthols **16** and a variety of substituted allylic alcohols **15** via toluene sulfonic acid-promoted series of cyclization reactions in dichloromethane to result in a library of dihydronaphthofurans **17** in moderate to excellent yields (48%–99%). The developed methodology involved the TsOH-promoted [3 + 2] cycloaddition reaction to generate intermediate **A**, which was subjected to an intramolecular addition reaction with a subsequent 1,2-hydride shift to form intermediate **C**. The resulting intermediate **C** then afforded the target molecules **17** in efficient yields with confined diastereoselective ratios (more than 20:1) on the subsequent addition of a nucleophile along with the removal of a proton. (Scheme 2).

**SCHEME 3:**
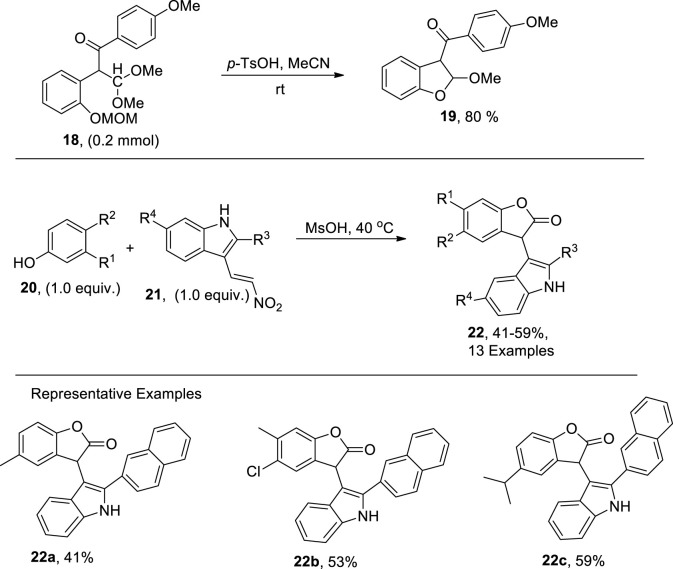
Synthesis of dihydrobenzofurans **19** and **22** using a Bronsted acid as the catalyst.

Nakamura et al. (2022) established the chalcone rearrangement strategy by treating chalcone **18** utilizing *p*-toluene sulfonic acid in acetonitrile to synthesize 2,3-dihydrobenzofuran **19** in efficient yields (Scheme 3). The dihydrobenzofuran derivatives obtained via mentioned protocol were then transformed into two different types of benzofuran derivatives by employing varied reaction conditions. Slightly acidic or basic conditions gave 3-acylbenzofurans, while conversion using *p*-toluene sulfonic acid resulted in the synthesis of 3-formylbenzofuran heterocycles.

In organic synthesis, various types of condensation reactions are applied to furnish several heterocycles. Grishin et al. (2022) proposed a novel and facile synthetic protocol for the synthesis of benzofuran derivatives via a Bronsted-acid-promoted condensation reaction between phenols **20** and nitrovinyl-substituted indoles **21**. The reaction was carried out in the presence of MsOH in 4 mL at 40°C to obtain high yields of benzofuran derivatives **22** (Scheme 3).

**SCHEME 4:**
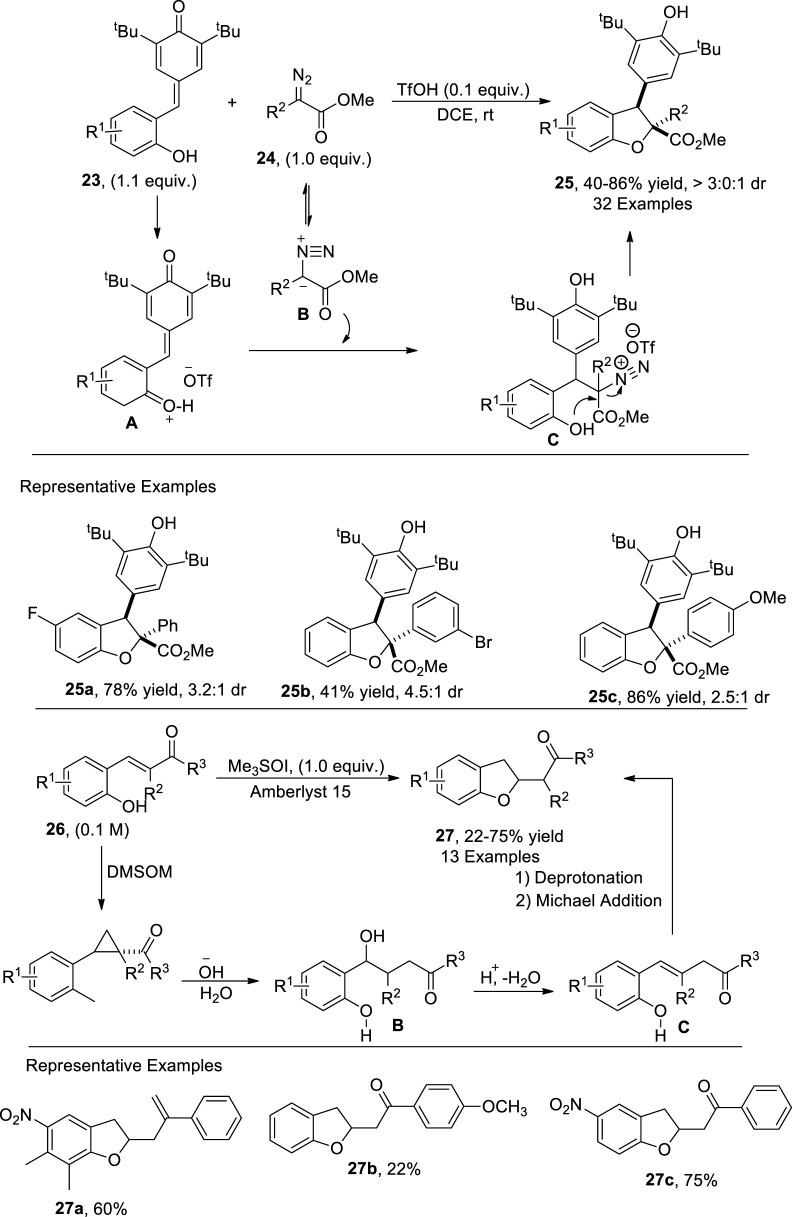
Synthesis of dihydrobenzofurans **25** and **27** using a Bronsted acid as the catalyst.

Bronsted acid-catalyzed [4 + 1] annulation reactions have also been applied to furnish heterocyclic scaffolds (Das et al., 2023). Lu et al. (2021) afforded the synthesis of 2,3-dihydrobenzofuran derivatives **25** (comprising of a quaternary C center) via a metal-free, TfOH-catalyzed protocol. In their developed synthetic route, *p*-quinone methides **23** were reacted with α-aryl diazoacetates **24** via [4 + 1] annulation. The suggested mechanistic insights revealed that the oxonium ion intermediate **A** was formed from **23** after interaction with TfOH followed by the generation of phenol-substituted intermediate **C** by undergoing a Michael addition with diazoester **B**. This intermediate **C** then underwent nucleophilic substitution reaction to form the desired products **25** in moderate to high yield (40%–86%) with significant diastereoselectivity (3:0:1 *dr*) (Scheme 4).

Corey–Chaykovsky reactions are carried out to achieve the synthesis of cyclopropanes, aziridine, and oxiranes by treating sulfur ylides with alkenes, imines, and carbonyl compounds, respectively (Heravi et al., 2016). Fadeev et al. (2022) accomplished a concise and efficient methodology for the preparation of a library of 2,3-dihydrobenzofuran scaffolds **27** by utilizing an extended Corey–Chaykovsky reaction. In their optimized strategy, 2-hydroxychalcones **26** were used as starting materials, which underwent further transformations in the presence of a Brønsted acid (Amberlyst 15) and Corey ylide (Me_3_SOI) to form 2,3-dihydrobenzofurans **27** in low to high yields (22%–75%). The recommended mechanistic details inferred that 2-hydroxychalcone **26** reacted with Me_3_SOI to form a reactive cyclopropane intermediate **A**, which was further treated with an alkali solution, followed by subsequent dehydration to give intermediate **B**. The intermediate **B** upon intramolecular *oxa*-Michael reaction resulted in 2,3-dihydrobenzofurans **27** (Supplementary Scheme 4).

### 2.2 Bronsted–Lewis acid-induced synthesis of dihydrobenzofurans

**SCHEME 5:**
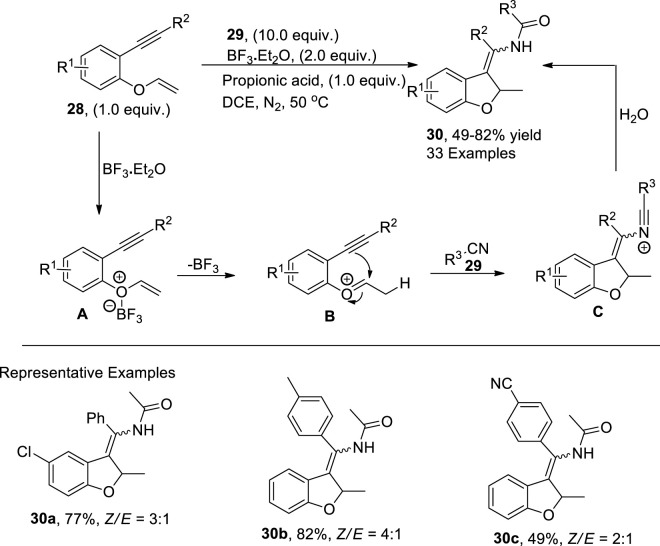
Synthesis of dihydrobenzofuran **30** using a Bronsted–Lewis acid as the catalyst.

Chen et al. (2023) reported a combination of Lewis and Bronsted acid (boron trifluoride diethyletherate and propionic acid)-catalyzed synthesis of 3-enamide-substituted dihydrobenzofurans **30** via the reaction of 1,6-enynes **28** and nitriles **29** in DCE under a nitrogen atmosphere. This synthetic approach has a diverse substrate scope with the chemoselective unistep construction of C–C and C–N bonds to facilitate the moderate to high yields of target molecules, that is, 49%–82%. As per the documented mechanistic details, the Bronsted acid interacted with 1,6-enyne **28** to generate an intermediate **A**, which underwent an electrophilic addition reaction with propionic acid to afford the oxonium intermediate **B**. In the next step, intermediate **B** underwent cyclization and reacted with nitrile **29** to give another intermediate **C**. Upon hydrolysis of intermediate **C**, desired 2,3 disubstituted dihydrobenzofurans **30** were attained (Scheme 5).

### 2.3 Photocatalytic synthesis of dihydrobenzofurans

**SCHEME 6:**
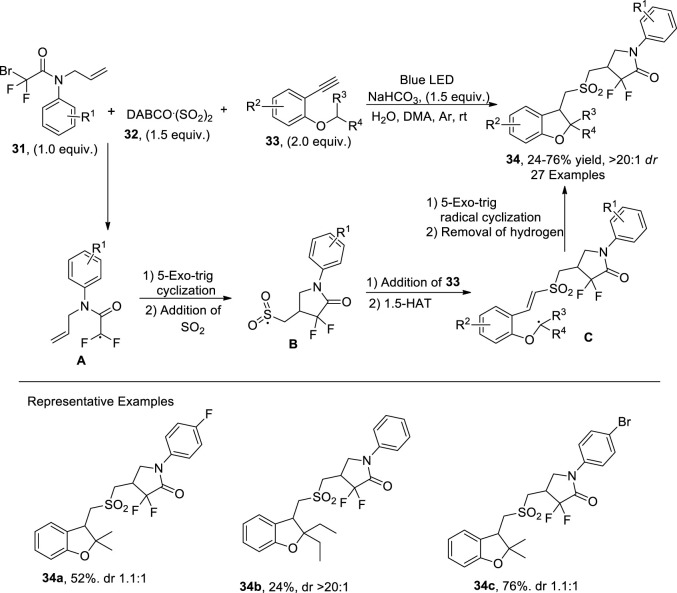
Synthesis of dihydrobenzofuran **34** by a photocatalytic reaction.

Visible light-catalyzed reactions are widely utilized to carry out profitable organic syntheses (Vellakkaran and Hong, 2021; Mueller et al., 2015; Chen et al., 2012). Shen et al. (2022) designed a visible light-mediated multicomponent cascade protocol for the synthesis of difluoroamidosulfonylated dihydrobenzofurans **34** via condensation of *N*-allylbromodifluoroacetamides **31**, terminal alkynes **33**, and DABCO^.^ (SO_2_)_2_ **32**. The conditions employed for this tandem reaction incorporated NaHCO_3_ (as the base) and DMA (as the solvent) to obtain good yields of dihydrobenzofurans. In their synthetic practice, a plausible mechanism involved the combination of allylbromodifluoroacetamides **31** and DABCO^.^ (SO_2_)_2_ **32** followed by visible light irradiation to generate radical intermediate **A**. The intermediate **A** then swiftly underwent 5-exo trig radical-mediated cyclization, proceeded by entangling sulfur dioxide to acquire the difluoroamidosulfonyl radical **B**. The intermediate **B** was then made to react with terminal alkynes **33** followed by 1,5-hydrogen atom transfer (HAT), which caused the emergence of another radical intermediate **C**. Later, the intermediate **C** encountered additional 5-exo trig radical annulation in sequence with the addition of hydrogen atom to secure dihydrobenzofurans **34** in efficient yields (Scheme 6).

**SCHEME 7:**
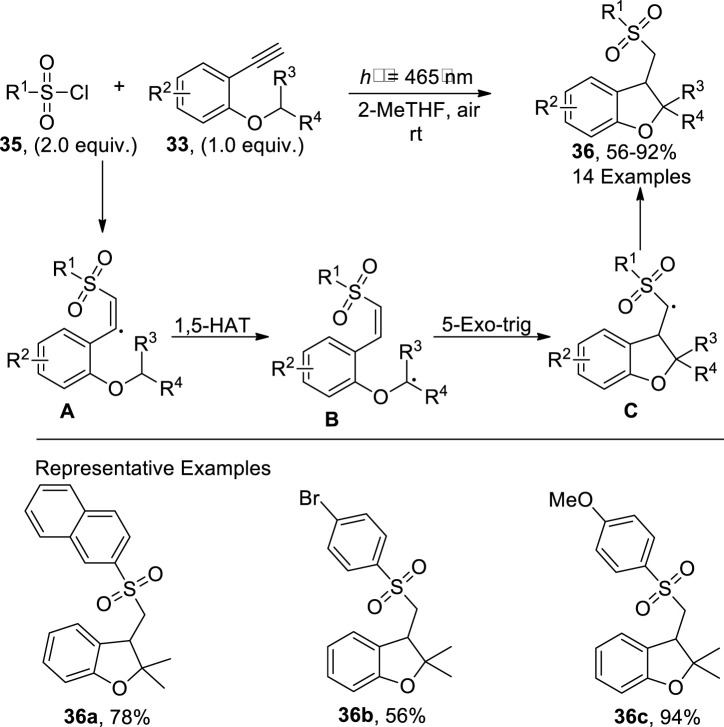
Synthesis of dihydrobenzofuran **36** by a photocatalytic reaction.

Another synthetic approach involving the visible light-mediated synthesis of dihydrobenzofurans was presented by Li et al. (2022). In this approach, substituted alkynyl ethers **33** were made to react with a diverse variety of sulfonyl chlorides **35** in the presence of photocatalyst and 2-MeTHF solvent without any additive to accomplish sulfonyl-substituted dihydrobenzofuran derivatives **36** in a 56%–93% yield range. The reaction mechanism was assumed to involve the excitation of alkynyl ethers **33** by visible light, followed by quenching with sulfonyl chloride **35** to give radical intermediate **A**, which upon intramolecular 1,5-hydrogen atom transfer gave rise to radical intermediate **B**. The radical intermediate **B** was then believed to undergo 5-exo trig (radical) cyclization to develop intermediate **C**, which led to the procurement of dihydrobenzofuran derivatives **36** after the abstraction of proton (Scheme 7).

**SCHEME 8:**
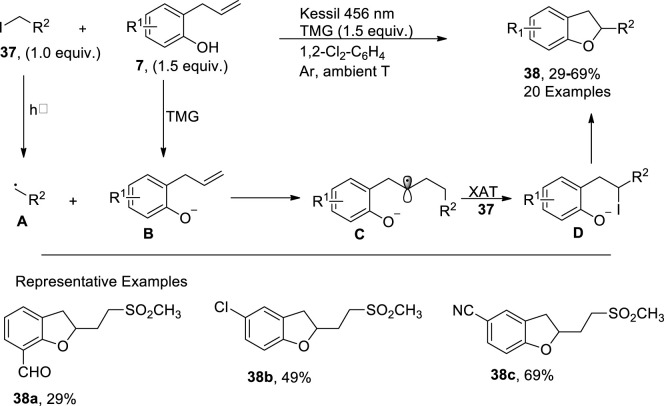
Synthesis of dihydrobenzofuran **38** by a photocatalytic reaction.

In 2023, Corti et al. (2023) developed a facile and efficient photo-induced method for the synthesis of sulfonated 2,3-dihydrobenzofuran derivatives **38**. In their metal-free strategy, 2-allyphenol derivatives **7** were made to react with α-iodo sulfones/alkyl iodide **37** in the presence of 1,1,3,3-tetramethylguanidine (TMG used as a base) and 1,2-dichlorobenzene (as a solvent) within a short duration (i.e., 35 min). This methodology covered a broad substrate scope with low to high yield (29%–69%) of target molecules. The synthesis commenced from the generation of phenolate anions **B** from 2-allyphenol derivatives **7** that stimulated the α-iodo sulfones/alkyl iodide **37** to form radical precursor **A**, which further reacted with the alkene part of the **B** to proceed via the tandem atom transfer radical addition (ATRA) cycle. The ATRA cycle generated intermediate **D** through the halogen atom transfer of *in situ* intermediate **C**, which, upon the subsequent nucleophilic substitution reaction, synthesized the desired 2,3-dihydrobenzofuran derivative **38** (Scheme 8).

**SCHEME 9:**
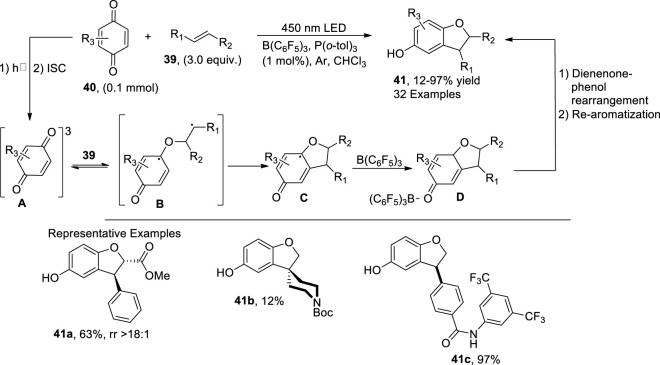
Synthesis of dihydrobenzofuran **41** by a photocatalytic reaction.

Maejima et al. (2023) envisioned a facile, one-pot, photocatalytic methodology for the synthesis of regioselective C_3_-substituted dihydrobenzofuran derivatives **41**. In this regard, *p*-benzoquinones **40** were reacted with substituted alkenes **39** in the presence of catalytic amounts of Lewis acid (LA) B(C_6_F_5_)_3_ and Lewis base P (*o*-tol)_3_. The mechanistic investigations revealed the initial excitation and intersystem crossing of *p*-benzoquinones to generate intermediate **A**, followed by radical addition to alkenes, thereby leading to intermediate **B**. The Paterno–Buchi reaction [2 + 2 cycloaddition] thus resulted in the formation of oxetane intermediate **C**, which, upon coordination with a Lewis acid, generated oxetane-Lewis acid intermediate **D** followed by the dienone-phenol rearrangement to form the C_3_-substituted dihydrobenzofuran derivatives **41**. High yields (94%) were achieved when the reaction was carried out in the presence of an LED source (450 nm) in chloroform (solvent) (Scheme 9).

**SCHEME 10:**
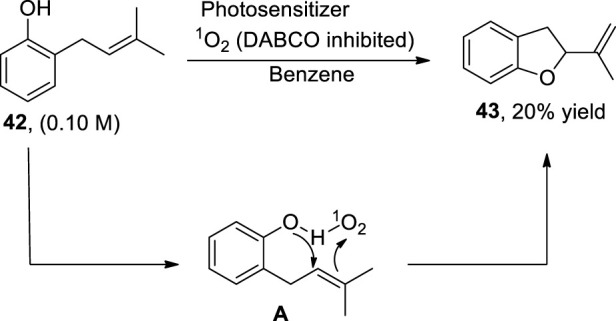
Synthesis of dihydrobenzofuran **43** by a photocatalytic reaction.

Being an integral structural constituent of various natural products, substituted dihydrobenzofurans have gained tremendous weight in organic synthesis. In this perspective, Jabeen et al. (2023) reported a methodology illustrating the role of a type II sensitized photooxidation and aprotic solvent toward the synthesis of 2,3-dihydrobenzofurans **43**. In this regard, *o*-prenyl phenol **42** was oxidized in the presence of type II oxygen species (^1^O_2_) and benzene (aprotic solvent) to synthesize 2,3-dihydrobenzofurans **43**. The aprotic solvent (benzene) was observed to increase the quenching rate by about ∼10-fold as compared to a protic solvent (methanol), thereby enhancing the reactivity of ^1^O_2_, which resulted in the formation of intermediate **A**. On decomposition, this intermediate **A** furnished the desired 2-(prop-1-en-2-yl)-2,3-dihydrobenzofurans **43** (up to 20%) and H_2_O_2_ (Scheme 10).

**SCHEME 11:**
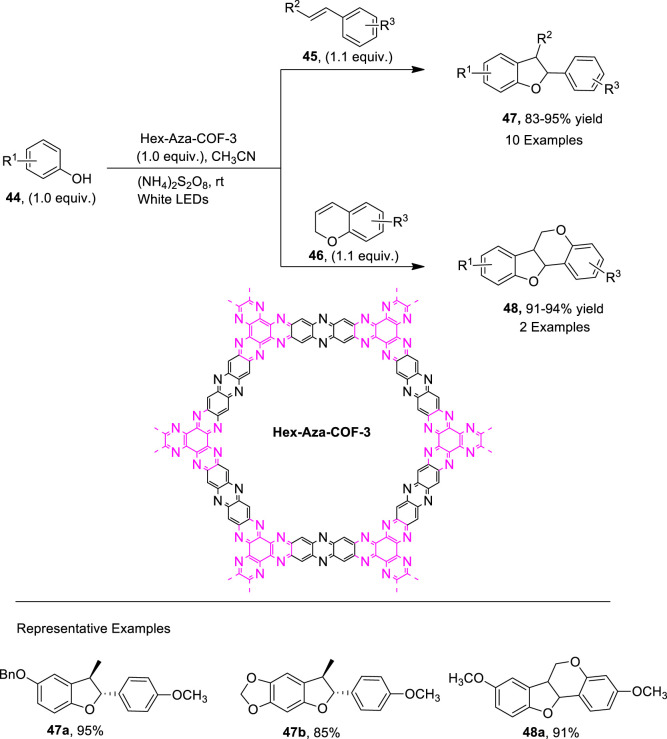
Synthesis of dihydrobenzofurans **47** and **48** by a photocatalytic reaction.

**FIGURE 3:**
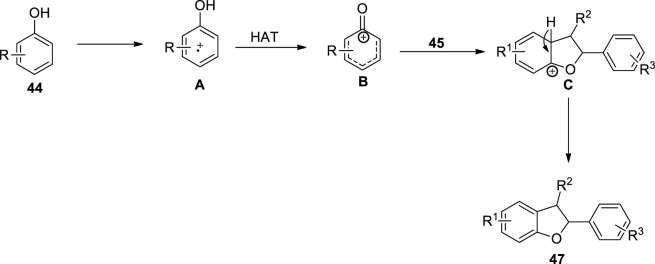
Proposed mechanism for the synthesis of dihydrobenzofuran **47** by a photocatalytic reaction.

Covalent organic frameworks (COFs) are important structural units that can be employed as convenient heterogeneous photocatalysts for many organic conversions, such as coupling reactions and olefin isomerization (Yu et al., 2021; Guo and Jiang, 2020; Ma et al., 2018). Parvatkar et al. (2023) proposed a photo-induced, metal-free [3 + 2] cycloaddition involving protocol for the efficient synthesis of dihydrobenzofuran derivatives. For this purpose, they utilized a previously synthesized heterogenous photo-active catalyst, that is, Hex-Aza-COF, and exploited it for the construction of dihydrobenzofuran motifs **47** and **48**. The reaction of substituted phenols **44** with olefins **45** and **46** via the oxidative cycloaddition in the presence of photocatalyst Hex-Aza-COF-3 white LEDs and (NH_4_)_2_S_2_O_8_ (oxidant) in acetonitrile (solvent) furnished the 2,3-dihydrobenzofuran **47** (up to 95% yield) and dihydro-6*H*-benzofuro [3,2-c]chromene **48** (up to 94% yield) (Scheme 11). The proposed mechanism for this reaction involved the light irradiation of the photocatalyst to obtain e^−^-h^+^ separation of charges, which further resulted in the oxidation of phenol to generate intermediate **A**. The intermediate **A** was then subjected to the hydrogen atom transfer (HAT) process to result in intermediate **B**, which furnished intermediate **C** upon a [3 + 2] cycloaddition reaction with substituted olefins **45**, which finally led to the desired dihydrobenzofurans via aromatization (Figure 3).

**FIGURE 4:**
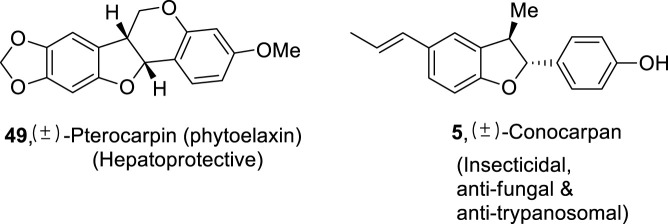
Structure of natural products constituting dihydrobenzofuran skeleton **5** and **49**.

Organic chemists are continuously progressing toward the total synthesis of natural products by using several synthetic routes (Faiz et al., 2017; Toure and Hall, 2009; Munawar et al., 2022). The dihydrobenzofuran derivatives synthesized using the above protocol were also applied as a precursor for the synthesis of naturally occurring compounds, namely, (±)-conocarpan **5** and (±)-pterocarpin **49** (Figure 4).

**SCHEME 12:**
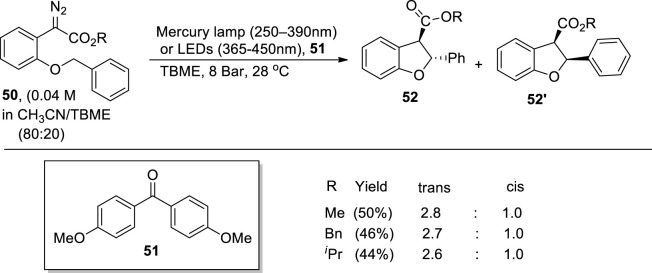
Synthesis of dihydrobenzofurans **52** and **52′** by a photocatalytic reaction.

O'Callaghan et al. (2023) reported a metal-free protocol for the synthesis of dihydrobenzofuran derivatives **52** and **52′** via C-H insertion, which was achieved by flow photolysis of aryldiazoacetate **50**. For this purpose, a photochemical chamber was utilized in which different wavelength filters (365–450 nm of LEDs or 250–390 nm of mercury lamp) with a pressure of 8 bar (generated via back pressure regulator) were employed in *tert*-butyl methyl ether (TBME) to achieve low to moderate yields (up to 50%) of desired products **52** and **52′** (substituted dihydrobenzofuran). Moreover, in the presence of a suitable photosensitizer **51** (i.e., 4,4′-dimethoxybenzophenone), enhanced diastereoselective products (i.e., trans isomer) were observed (Scheme 12).

**SCHEME 13:**
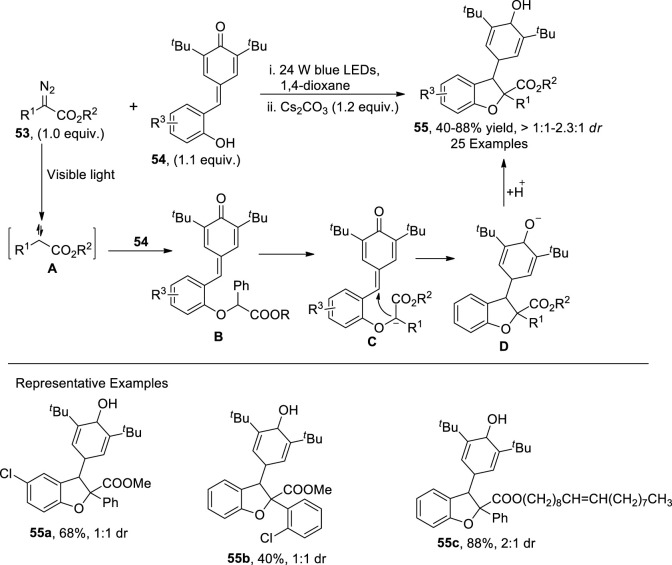
Synthesis of dihydrobenzofuran **55** by a photocatalytic reaction.

Zhou et al. (2021) put forward the visible light-mediated, base-catalyzed protocol for the synthesis of 2,3-dihydrobenzofurans **55** by making use of an O-H insertion reaction and cyclization. In their synthetic approach, diazo compounds **53** and substituted *para*-quinones **54** under the irradiation of blue LED and Cs_2_CO_3_ afforded the 2,3-disubstituted dihydrobenzofurans **55**. The proposed mechanism suggested that the diazo compounds **53** acted as a source of free carbene species **A**, which reacted with *p*-quinone **54** via O-H insertion to generate intermediate **B**. The intermediate **B** swiftly went through base-mediated deprotonation to furnish intermediate **C**. Finally, cyclization of **C**, followed by hydrogenation, generated the desired dihydrobenzofuran scaffolds **55** (Scheme 13).

### 2.4 Base-induced synthesis of dihydrobenzofurans

**SCHEME 14:**
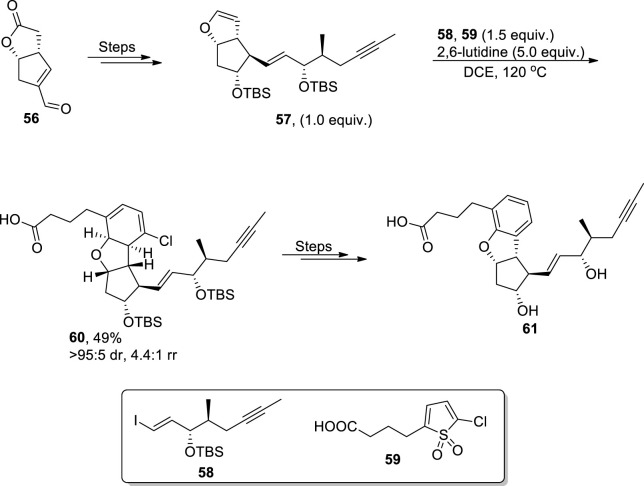
Synthesis of the dihydrobenzofuran derivative **61** by a base-induced reaction.

Among surplus synthetic pathways to achieve dihydrobenzofurans, base-promoted approaches have gained noteworthy significance (Medishetti et al., 2021). Wang et al. (2023) established the total synthesis of dihydrobenzofuran, which constitutes the anti-platelet and vasodilator drug Beraprost. They utilized the innovative protocol to access the dihydrobenzofuran core by treating enol ether **57** (accessed by subjecting enal lactone **56** to several steps) with substituted thiophene-1,1-dioxide **59** by carrying out an inverse-electron demand Diels–Alder reaction (IEDDA) followed by cheletropic elimination of sulfur dioxide, removal of the halogen, and aromatization in 2,6-lutidine (base) to afford the dihydrobenzofuran core. The dihydrobenzofuran core **60** was achieved in 49% yield with 4:4:1 rr (regioisomeric ratio). The dihydrobenzofuran core-substituted compound **60** was then reacted further over several steps to yield a biologically active drug **61** (Scheme 14).

**SCHEME 15:**
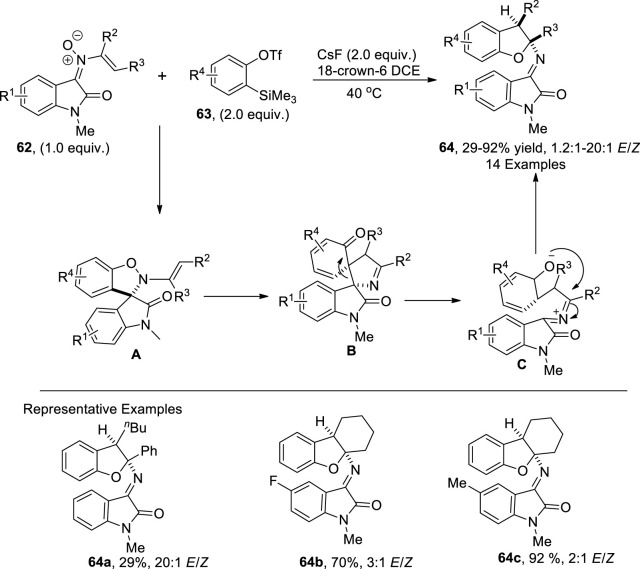
Synthesis of the dihydrobenzofuran derivative **64** by a base-induced reaction.

Cycloaddition reactions are widely utilized to construct diversely functionalized heterocycles, owing to their efficient high atom economic and high-yielding nature (Meloche and Ashfeld, 2017; Kobayashi and Jørgensen, 2002). In 2022, Yuan et al. (2022) developed a synthetic protocol for synthesizing 2,3-dihydrobenzofurans **64**. In their developed pathway, *N*-vinyl oxindole nitrones **62** were reacted with *o*-silylphenyl triflates **63** in the presence of CsF (base) to furnish desired polycyclic 2,3-dihydrobenzofurans **64** in low to high yields (29%–92%) with a notable *E*/*Z* ratio (20:1). The advocated mechanism inferred the employment of *o*-silylphenyl triflates **63** as aryne precursors, which underwent [3 + 2] cycloaddition with *N*-vinyl oxindole nitrones **62** to generate a cycloadduct **A**, which was further transformed into intermediate **B** via [3,3] rearrangement. Next, this intermediate was converted into desired 2,3-dihydrobenzofurans **64** by undergoing a retro-Mannich reaction and intramolecular cyclization (Scheme 15).

**SCHEME 16:**
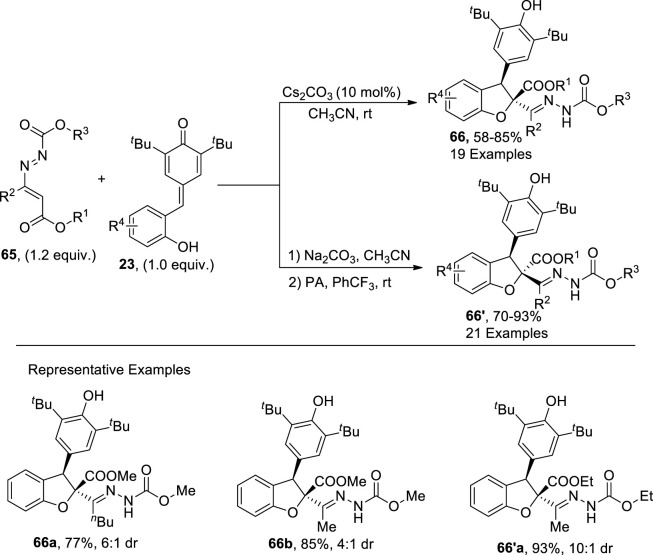
Synthesis of the dihydrobenzofuran derivative **66** and **66′** by a base-induced reaction.

In 2023, Guan et al. (2023) performed the very first [4 + 1] cycloaddition reaction to attain adjustable diastereoselective synthesis of dihydrobenzofurans **66**. They treated azoalkenes **65** (utilized as synthon of one carbon) with para quinone methides **23** via a diastereodivergent cycloaddition reaction. The cesium carbonate (base)-catalyzed [4 + 1] cycloaddition reaction between the given substrates resulted in less crowded thermodynamic target molecules **66** (in a 58%–85% yield range). However, when the reaction was performed by utilizing phosphoric acid (a Bronsted acid) as a catalyst, H-bonding linkages gave rise to kinetic products dominantly (in the 70%–93% yield range). Their synthetic protocol involving [4 + 1] cycloaddition proceeded via oxa-1,6-addition and 1,4-addition operations (Scheme 16).

**SCHEME 17:**
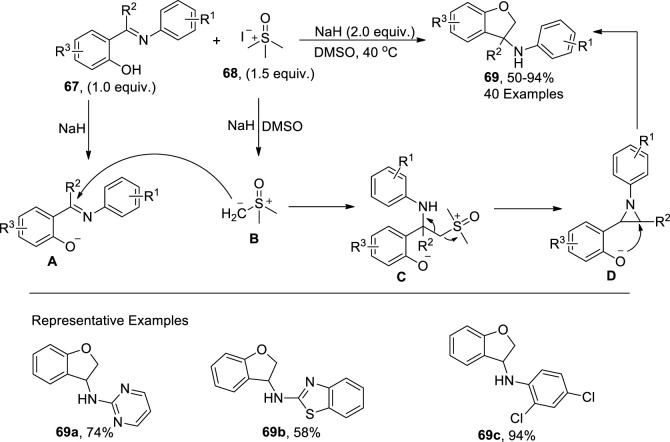
Synthesis of the dihydrobenzofuran derivative **69** by a base-induced reaction.

Fang et al. (2022) developed a green and effective base-mediated procedure to access 3-amino-2,3-dihydrobenzofurans via [4 + 1] cyclization of trimethylsulfoxonium iodide **68** and substituted 2-hydroxylimides **67** in moderate to excellent yields (50–94%). In the presence of a base (NaH), sulfur ylide **B** attacked anionic 2-hydroxylimide **A**, affording an intermediate **C**. The subsequent cyclization of the intermediate **C** gave rise to the aziridine formation **D**, followed by the intramolecular cyclization to acquire the desired 3-amino-2,3-dihydrobenzofurans **69** in DMSO (Scheme 17).

**SCHEME 18:**
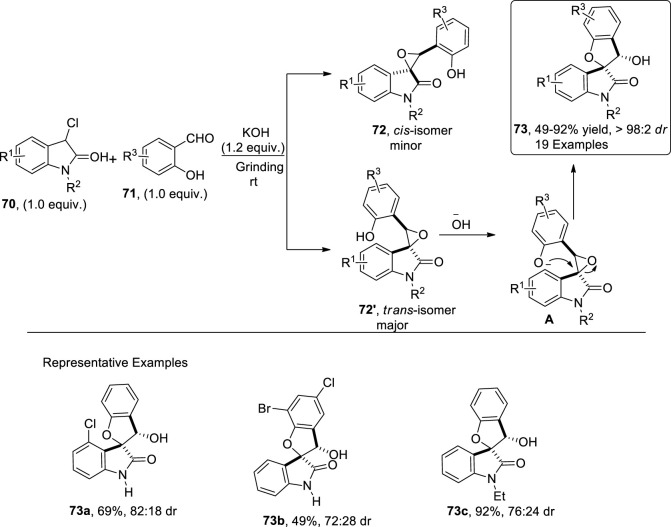
Synthesis of the dihydrobenzofuran derivative **73** by a base-induced reaction.

Epoxide ring opening reactions are of significant importance in the synthesis of diverse organic molecules (Faiz and Zahoor, 2016; Ahmad et al., 2018). Li et al.(2023) reported the synthesis of dihydrobenzofuran spirooxindole scaffolds **73** through a solvent-free grinding protocol. To accomplish this, they treated α-chlorooxindoles **70** with salicylaldehyde **71** in the presence of KOH via [4 + 1] cyclization to afford the desired product **73** in good to excellent yields (49%–92%) with notable diastereoselectivities (up to 98:2 *dr*). As per the proposed mechanistic details, enolate carbanion emerged by treating chlorooxindoles **70** with a Bronsted base (KOH), which then underwent Darzen-type epoxidation with salicylaldehyde **71** to generate two isomers, that is, *syn* **72** and the most probably *anti* isomer **72′** (more stable). Next, a base-mediated intramolecular furan ring-closing and epoxide ring-opening reaction of **72′** occurred, thereby leading to the formation of *syn*-2,3-dihydrobenzofuranspirooxindoles **73** (Scheme 18).

**SCHEME 19:**
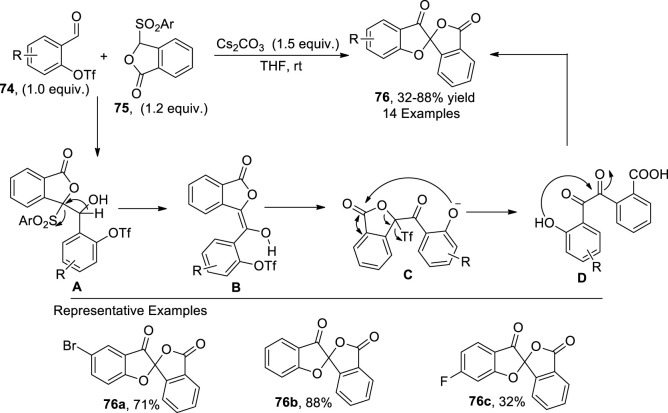
Synthesis of the dihydrobenzofuran derivative **76** by a base-induced reaction.

Nair et al. (2023) envisioned a base-mediated protocol for synthesizing isobenzofuranone-spiro-linked benzofuranone **76**. In their synthetic approach, formyl triflates **74** were made to react with sulfonylphthalide **75** via a Hauser–Kraus reaction ([4 + 4] annulation reaction) in the presence of Cs_2_CO_3_ (base) in THF to synthesize the desired dihydrobenzofuranones **76** in low to high yields (32%–88%). According to the plausible mechanism investigation, the phthalide anion generated from **75** attacked the carbonyl center of **77**, followed by the desulfonation to generate intermediate **B**, which, upon enolization and triflate capture, afforded an oxonium intermediate **C**. In the next step, the oxonium anion attacked the lactone carbon to form an eight-membered ring via the Dieckmann process, which underwent a ring-opening reaction with carbonate, followed by the elimination of CO_2_ to form the benzil intermediate **D**. Finally, dehydration of **D** leads to the formation of the benzofuranone scaffold **76** (Scheme 19).

**SCHEME 20:**
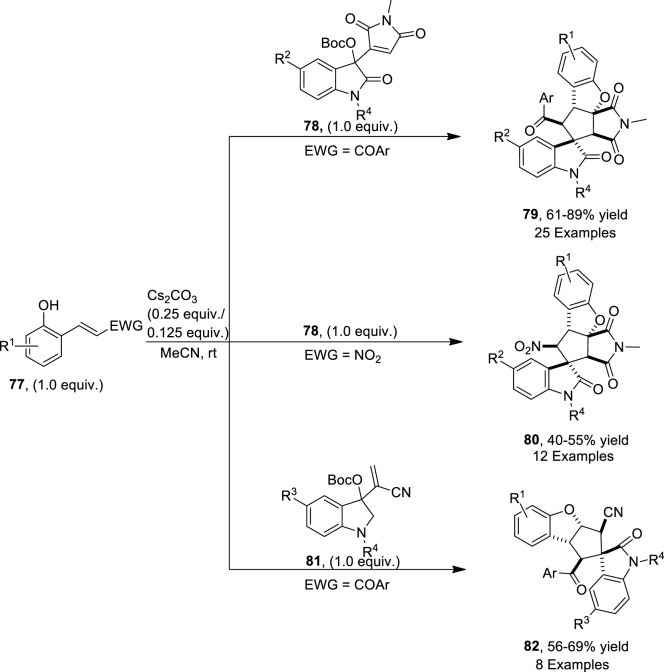
Synthesis of dihydrobenzofuran derivatives **79**, **80**, and **82** by a base-induced reaction.

**FIGURE 5:**
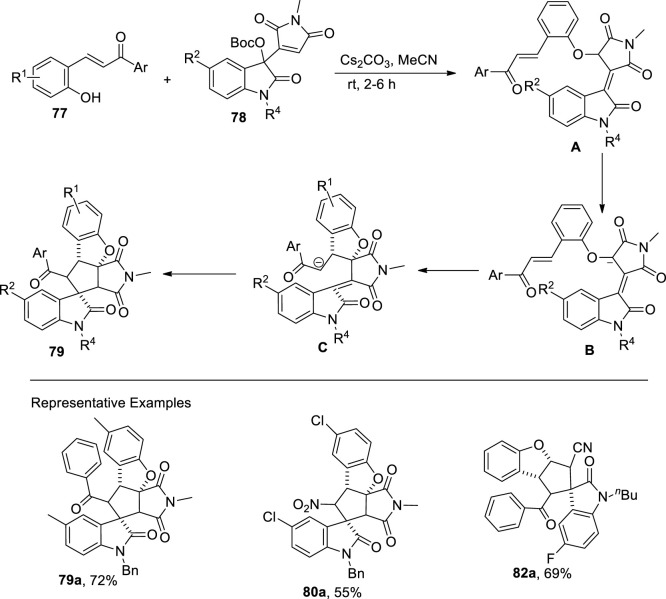
Proposed mechanism for the synthesis of the dihydrobenzofuran derivative **79** using a base-induced reaction.

Wang et al. (2022) disclosed a base that is a cesium carbonate-mediated reaction of β-nitro-ortho-hydroxystyrenes (EWG = NO_2_)/ortho-hydroxychalcones (EWG = COAr) **77** with Morita−Baylis−Hillman (MBH) maleimides **78** and nitriles **81** of isatin via cascade annulation to access dihydrobenzofuran-spirooxindole motifs **79**, **80**, and **82**, respectively. The reaction was carried out in MeCN (as a solvent) for approximately 2 h to obtain a maximum yield of 89%. In this domino reaction, phenolates of hydroxychalcones/hydroxystyrenes **77** attacked at the α-position of MBH maleimides/nitriles **78** or **81** to result in the formation of an intermediate, which underwent a double Michael addition reaction to give final products **79**, **80**, and **82**, respectively (Scheme 20) (Figure 5).

Chen et al. (2021) proposed an efficient, metal-free cascade approach for constructing dihydrobenzofuran skeletons. In their novel approach, an ortho-allyloxy benzenediazonium salt reacted with thiophenols in the presence of DABCO (1,4-diazabicyclo [2.2.2]octane), which was used as an additive and an organic base, to afford desired heterocyclic products in moderate to high yields (63%–83%). The DABCO-catalyzed thiolate anion interacted with diazonium salt to generate diazo sulfide **B**, followed by thermal homolysis to give sulfenyl radical **C** and intermediate **D**. The intermediate **D** then proceeded through intramolecular 5-*exo* cyclization between the aromatic radical and the double bond, followed by reaction with sulfenyl radical **C** to furnish 2,3-disubstituted dihydrobenzofuran derivatives (Supplementary Scheme 1).

### 2.5 Catalyst-free synthesis of dihydrobenzofurans

Catalyst-free synthesis is a proficient and green approach that has been extensively applied in organic synthesis to avoid the use of expensive catalysts (Gawande et al., 2013). Das et al. (2022) subjected allyl-linked diazonium salts with substituted vinyl boronic acid and DABSO (1,4-diazabicyclo [2.2.2]octane bis(sulfur dioxide) adduct) under catalyst-free conditions utilizing KHCO_3_ in an optimized ratio of dimethylformamide and acetone (9:1) to afford sulfonylated dihydrobenzofuran derivatives in efficient yields (70%–80%) (Supplementary Scheme 2). Another example of catalyst-free synthesis was reported by Rao et al. (2023). They initially treated substituted salicylaldehydes with chloroacetonitrile in the presence of sodium carbonate and NaI in dimethyl formamide to obtain benzaldehyde derivatives. These derivatives were then reacted with substituted anilines in a catalyst-free approach to afford remarkable yields (80%–90%) of benzofuran derivatives (Supplementary Scheme 3).

Bisag et al. (2021) experimented with another catalyst-free protocol for the synthesis of 2,3-dihydrobenzofuran scaffolds. In their synthetic approach, substituted salicylaldehydes were reacted with sulfoxonium ylide in non-catalyst conditions to afford dihydrobenzofurans in high yields (80%–89%) in CH_2_Cl_2_. Sulfur ylide attacked the carbonyl carbon of substituted salicylaldehydes, ensuing a proton transfer reaction that resulted in the emergence of intermediate **B**. The intermediate **B**, upon dehydration, generated intermediate **C**. The desired products were finally obtained after a [4 + 2] cyclization reaction between ylide and intermediate **C** (Supplementary Scheme 4).

Another catalyst-free synthesis of substituted dihydrobenzofurans was developed by Zhou et al. (2022) recently via a [3 + 2] cyclization of unsaturated imines and iodine-substituted phenols in the presence of base (Supplementary Scheme 5). The reaction mechanism was assumed to move forward with the formation of intermediate **A**, which underwent C-I bond breakage followed by tautomerization and an addition reaction with iodine radical to give diradical intermediate **D**. The intermediate **D** then resulted in the synthesis of target molecules as a result of subsequent cyclization and hydrolysis. (Supplementary Scheme 5). Considering the deadly effects of cancer, researchers are continuously working to develop novel anti-cancer agents (Emami and Dadashpour, 2015; Irfan et al., 2022). The dihydrobenzofuran derivatives obtained via the above-mentioned protocol were utilized to synthesize anti-cancer agents and α-glucosidase inhibitors (Supplementary Figure 1).

### 2.6 Iodine-induced synthesis of dihydrobenzofurans

Iodine-mediated synthesis of organic molecules has earned significant weight owing to its economical nature and correspondence with green chemistry criteria (Samanta and Mondal, 2021; Banerjee et al., 2006). Cheng et al. (2023) envisioned an efficient, iodine-catalyzed protocol for generating 2,3-dihydrobenzofuran units. In their established protocol, chalcones and isobutyraldehyde were reacted in the presence of I_2_ (catalyst) to procure the desired dihydrobenzofuran motifs in good yield (67%). This synthetic pathway proceeds at ambient reaction conditions and covers a diverse range of functional groups (Supplementary Scheme 6).

Chen et al. (2023) disclosed a highly efficacious and metal-free transformation for synthesizing 3-aryl-2,3-dihydrobenzofurans. In their established pathway, *ortho*-hydroxystilbenes reacted with alcohol via cyclization and the aryl migration (CAM) process in the presence of an iodine oxidant in ethanol. *o*-Hydroxystilbenes were activated and converted to an iodonium ion intermediate **A** in the presence of an oxidant (I_2_ or ICI), followed by the subsequent removal of HI and aryl migration in the acidic medium to generate intermediate **D**. The intermediate **D** was then subjected to alcohol to synthesize target molecules in low to excellent yields (19%–92%) (Supplementary Scheme 7).

Azevedo et al. (2023) developed an eco-friendly approach involving solvent-free synthesis of chalcogenyl-based dihydrobenzofuran derivatives. In their green methodology, substituted allylphenols were treated with diaryl diselenides under metal-free, microwave-irradiated neat conditions, utilizing catalytic iodine with DMSO as an oxidant. The resulting dihydrobenzofuran derivatives were then evaluated for MAO-B inhibitory potential. The MAO-B inhibitory activity of synthesized dihydrobenzofurans gave indication about their utilization against Alzheimer’s and Parkinson’s diseases (Supplementary Scheme 8). The suggested mechanism inferred the reaction of diselenides with iodine using microwave irradiation to form electrophilic species **A**. In the next step, **A** coordinated with allylphenols to create the chalcogeniranium intermediate **B**, which opened via phenoxide ion to result in the required dihydrobenzofurans (Supplementary Figure 2).

### 2.7 Electrocatalytic synthesis of dihydrobenzofurans

Electrocatalytic reactions have been observed to be feasible and environmentally benign synthetic pathways to accomplish the synthesis of several organic compounds (Hammerich and Speiser, 2015; Nikishin et al., 1988). Elinson et al. (2021) proposed the synthesis of benzofuran derivatives by employing an electrolysis-mediated one-pot reaction of *N*,*N*-dimethylbarbiturate cyclic diketone and substituted aryl aldehydes. The reaction mechanism was thought to involve deprotonation, Knoevenagel condensation, Michael addition, bromination, and cyclization to yield dihydrobenzofurans. Another electrolysis-mediated facile approach leading to the synthesis of benzofuran heterocycles, including methanol and sodium bromide in reaction media, was presented by Ryzhkov et al. (2021). This novel methodology involving the cyclization of benzylidenebarbiturates and cyclic diketones in selected optimal conditions gave high yields of target molecules (72%–85%). As per the mechanistic details, a dienone anion (obtained by reaction with methoxide ion) was added to a cyclic diketone to give intermediate **A**, which was then subjected to treatment with bromine followed by exposure to methoxide ion, thereby resulting in intermediate **C**. The generated intermediate then underwent cyclization to afford corresponding dihydrobenzofuran derivatives (Supplementary Scheme 9; Figure 3).

### 2.8 Organocatalyzed synthesis of dihydrobenzofurans

Owing to their significant correspondence with green chemistry criteria, organocatalytic reactions have also flourished as efficient catalytic systems in organic transformations (Raj and Singh, 2009; Moyano and Rios, 2011). Xiang et al. (2021) proposed an efficient, organocatalyzed protocol for the synthesis of asymmetric dihydrobenzofurans via a Michael addition/hemiketalization reaction. In their novel methodology, substituted quinones were reacted with hydroxymaleimides in the presence of a chiral organocatalyst via a Michael addition followed by aromatization and hemiketalization to synthesize target molecules in moderate to excellent yields (68%–97%) with high enantiomeric (63:37-95:5 *er*) and diastereomeric ratios (>99:1 *dr*) (Supplementary Scheme 10).

Quinine-derived bifunctional squaramides were used by Susam et al. (2022) as active organocatalysts for the efficient synthesis of stereoselective dihydrobenzofurans and dihydronaphthofuran derivatives. In their synthetic approach, they used domino-type Friedel–Crafts/S_N_2 reactions to achieve the desired products in low to excellent yields (33%–95%) with remarkable enantioselectivity values (>99% *ee*). (*Z*)-α-Bromonitroalkenes were made to react with β-naphthols, α-naphthols, and substituted phenols in the presence of DABCO (used as a base) to achieve dihydronaphthofurans and dihydrobenzofurans, respectively (Supplementary Scheme 11).

### 2.9 Synthesis of dihydrobenzofurans by utilizing miscellaneous strategies

Asymmetric synthesis has been crucial in organic synthesis as various advanced and biologically important materials have chiral functionality (Taggi et al., 2003; Tsuji et al., 2018; Zbieg et al., 2012). *N*-Phosphinyl and *N*-phosphonyl imine acted as group-assisted purification (GAP) reagents for asymmetric synthesis (An et al., 2015). These GAP reagents have been used to prepare crystalline and solid products instead of oily and semi-solid products to avoid impurities. Rouh et al. (2022) reported the asymmetric synthesis of functionalized dihydrobenzofuran derivatives via an aggregate-based system. In their novel methodology, GAP reagents, that is, *N*-phosphonyl imines, were used to achieve the desired products in good to high yields (59%–70%) with excellent purity and diastereoselectivity values (77:23 *S*/*R*). Salicyl *N*-phosphonyl imines and dialkyl bromomalonates were reacted in different cosolvent ratios (THF:EtOH) and K_3_PO_4_ to furnish dihdrobenzofuran derivatives. When the ratios of these cosolvents were changed, the diastereomeric ratios of the products also changed. It was observed that with the increased amounts of ethanol in the THF:EtOH aggregate system, a high yield of the (*R*)- isomer was obtained over the (*S*)-isomer, and there was a reversal of results when ^*i*^Pr was used instead of EtOH. The formation of these aggregates of asymmetric starting material was measured by aggregation-induced polarization (an analytical technique) (Supplementary Scheme 12).

Scherübl et al. (2021) described the TEMPO (2,2,6,6-tetramethyl piperidine 1-oxyl)-promoted reaction of *m*-allyloxy aryl triflates (benzyne precursors) to synthesize dihydrobenzofuran derivatives by using cesium fluoride and 18-crwon-6-ether in n-hexane. TEMPO (acting as a stable radical) was added to *o*-substituted benzynes, thereby acting as a radical acceptor to generate aryl radicals. The generated radicals underwent 5-*exo*/6-*endo* cyclization with subsequent TEMPO-trapping to afford desired cyclic products in low to moderate yields (30%–62%) (Supplementary Scheme 13).

Roldan et al. (2023) demonstrated the influence of persistent radicals to avail the dihydrobenzofuran derivatives. For this purpose, they reacted persistent quinone methide radicals with phenols to afford transient phenoxy radicals. The transient and persistent radicals then underwent a cross-coupling reaction to result in pendant phenols and quinone methides, whose cyclization then furnished the synthesis of trans-2,3-diaryldihydrobenzofurans. The mechanism of this reaction was supposed to move forward by C–C bond formation, tautomerization, and cyclization to yield target molecules in efficient yields. This radical involving methodology provides an efficient pathway toward synthesizing naturally occurring stilbenoid, that is, resveratrol (Supplementary Scheme 14).

A metal-free synthetic route toward the procurement of dihydrobenzofuran derivatives was reported by Liu et al. (2022). They treated alkenyl-substituted phenols with sodium halides using K_2_S_2_O_8_ (as a promoter) in acetonitrile to obtain dihydrobenzofurans (Supplementary Scheme 15).

Another stereoselective approach to furnish dihydrobenzofurans was put forward by Kumari et al. (2023). In their stereoselective synthetic pathway, α-bromoketones were treated with pyridine-substituted Merrifield resin (promoter) in acetonitrile to generate pyridinium ylide **A**. The pyridinium ylides were then subjected to react with substituted *o*-hydroxychalcones via a [4 + 1] cyclization reaction using triethylamine as a base. As a result, chiral dihydrobenzofurans were attained in good to excellent yields (70%–92%). The formal [4 + 1] cyclization reaction was proposed to proceed via the conjugate addition of pyridinium ylide to substituted hydroxy chalcones followed by the attack of the nucleophilic oxygen to construct a carbon-oxygen bond, thus eliminating pyridine to yield the target molecules (Supplementary Scheme 16).

Röser et al. (2022) proposed a protocol for the synthesis of dihydrobenzofuran derivatives from β-ketolactone via the decarboxylation-cycloetherification method. The synthesis was accomplished by using a catalytic amount of quaternary ammonium iodides along with H_2_O_2_ (oxidant) in CH_2_Cl_2_ (solvent). This synthetic methodology covers a broad spectrum of substrates with good to excellent yields (57%–93%) under ambient reaction conditions. Cycloetherification is done *in situ* by the formation of ammonium hypoiodite from quaternary ammonium iodides. This synthesis was carried out by using β-ketolactone, affording a phenolic intermediate **A** that underwent oxidative cycloetherification to generate an intermediate **B**, which, upon further cyclization, resulted in the desired dihydrobenzofurans (Supplementary Scheme 17).

Lacuna et al. (2022) reported the utilization of the Pauson–Khand reaction by carrying out the cyclization of alkynylphenyl vinyl ethers. This less-time-consuming synthetic protocol resulted in efficient synthesis of numerous benzofuran derivatives in isolated yields (Supplementary Scheme 18). Kamitanaka et al. (2021) established the tetrabutylammonium triflate-catalyzed preparation of 2-oxygenated dihydrobenzofuran derivatives via [3 + 2] coupling. In their synthetic approach, quinone monoacetals (1.0 equiv.) reacted with vinyl ethers (2.0 equiv.) in HFIP utilizing ^*n*^Bu_4_NOTf (0.2 equiv.) to furnish dihydrobenzofurans in moderate to excellent yield range (45%–99%). Quinone monoacetals interacted with HFIP to form a charged intermediate **A**, followed by alkoxy group elimination and reaction with vinyl ethers at the α-carbon of the carbonyl group to generate intermediate **B**. This intermediate **B** then underwent aromatization to afford 2,3-dihydrobenzofurans (Supplementary Scheme 19).

Hellwig et al. (2021) used a versatile approach for the synthesis of 2,3-dihydrobenzofuran selenides. In their synthetic methodology, diorganyl dichalcogenides and 2-allylphenols were reacted in the presence of potassium peroxymonosulfate (Oxone^®^) as an effective oxidizing agent in MeCN. The proposed mechanistic insights unveiled that the alkyl diselenides were made to interact with Oxone^®^ to form electrophilic selenium species **A**, which further reacted with 2-allylphenols to generate seleniranium intermediate **B**. The intermediate **B** then underwent intramolecular oxyselenocyclization and deprotonation to afford the desired 2,3-dihydrobenzofuran with a moderate to high yield range (45%–85%) (Supplementary Scheme 20).

## 3 Conclusion

In summary, we have outlined novel transition metal-free synthetic strategies with mechanistic insights to achieve the construction of the dihydrobenzofuran nucleus, reported within the last 3 years (2021–2023). With respect to this, Bronsted acid-mediated, Lewis acid-induced, photocatalyzed, base-catalyzed, iodine-promoted, electrocatalytic, and organocatalyzed synthetic transformations have been discussed. In addition to the utilization of earlier mentioned proficient catalysts, catalyst-free synthesis of dihydrobenzofurans has huge implications. These methodical approaches involve various annulation, insertion, and cycloaddition reactions, that is, [3 + 2] annulation, [4 + 1] annulation, [4 + 4] annulation, [4 + 1] cycloaddition, C-H insertion, 5-exo-trig cyclization, and condensation reactions to carry out the synthesis of these biologically active heterocyclic scaffolds. We envisage that our review will urge the researchers to escalate these developments to acquire dihydrobenzofuran derivatives, thereby gauging their role in medicinal chemistry.
