# Supplementary material for: Novel transition metal-free synthetic protocols toward the construction of 2,3-dihydrobenzofurans: a recent update
Source: Front Chem. 2024 Dec 13;12:1470861. doi: 10.3389/fchem.2024.1470861 (PMC11672212; doi:10.3389/fchem.2024.1470861)
Supplement: Supplementary file 1 [file DataSheet1.docx]

**Supplementary File**

**Novel Transition Metal Free Synthetic Protocols Towards the Construction of 2,3-Dihydrobenzofurans: A Recent Update**

**Figure 1:** Structure of dihydrobenzofuran constituting bioactive compounds.

**Figure 2:** Proposed mechanism for the synthesis of dihydrobenzofuran derivatives by iodine induced approach.

**Figure 3:** Proposed mechanism for the synthesis of dihydrobenzofuran derivatives **114** by electrolysis induced approach.

**Scheme 1:** Synthesis of dihydrobenzofuran derivatives by using base induced reaction.

**Scheme 2:** Synthesis of dihydrobenzofuran derivatives by catalyst free approach.

**Scheme 3:** Synthesis of dihydrobenzofuran derivatives by catalyst free approach.

**Scheme 4:** Synthesis of dihydrobenzofuran derivatives by catalyst free approach.

**Scheme 5:** Synthesis of dihydrobenzofuran derivatives by catalyst free approach.

**Scheme 6:** Synthesis of dihydrobenzofuran derivatives by iodine induced approach.

**Scheme 7:** Synthesis of dihydrobenzofuran derivatives by iodine induced approach.

**Scheme 8:** Synthesis of dihydrobenzofuran derivatives by iodine induced approach.

**Scheme 9:** Electrolysis induced one-pot synthesis of dihydrobenzofuran heterocycles.

**Scheme 10:** Organocatalyzed synthesis of dihydrobenzofuran heterocycles.

**Scheme 11:** Organocatalyzed synthesis of dihydrobenzofuran heterocycles.

**Scheme 12:** Aggregate-induced synthesis of dihydrobenzofurans.

**Scheme 13:** TEMPO mediated synthesis of dihydrobenzofurans.

**Scheme 14:** Synthesis of dihydrobenzofuran by involving the effect of persistent radicals.

**Scheme 15:** Synthesis of dihydrobenzofurans by involving the effect of persistent radicals

**Scheme 16:** Synthesis of dihydrobenzofurans by using Merrifeld resin as catalyst.

**Scheme 17:** Synthesis of dihydrobenzofurans via decarboxylation-cycloetherification method

**Scheme 18:** Synthesis of dihydrobenzofurans by employing Pauson-Khand reaction.

**Scheme 19:** Tetrabutylammonium triflate catalyzed synthesis of dihydrobenzofurans.

**Scheme 20:** Synthesis of dihydrobenzofurans via oxyselenocyclization.
